# Scalable, modular whole-slide imaging pipelines for low-resource settings: A practical framework and open notebook implementation

**DOI:** 10.1016/j.jpi.2026.100692

**Published:** 2026-07-18

**Authors:** Alfred Githuka, Ulysses G.J. Balis, Ye Chan Kim, Eileen M. Weinheimer-Haus, Christopher L. Williams, Jerome I. Cheng, Kelou Yao, John Blau, Jessica A. Baker, Priscilla Njenga, Winny Chepkemoi, Robert K. Parker, Akbar K. Waljee, Shahin Sayed, Mansoor N. Saleh

**Affiliations:** a Department of Hematology-Oncology, Aga Khan University Hospital Nairobi, Nairobi, Kenya; b Department of Pathology, University of Michigan Health System, Ann Arbor, MI, USA; c Department of Learning Health Sciences, University of Michigan Medical School, Ann Arbor, MI, USA; d Center for Global Health and Equity, University of Michigan, Ann Arbor, MI, USA; e Department of Pathology, University of Oklahoma Health Sciences Center, Oklahoma City, OK, USA; f Department of Pathology and Laboratory Medicine, Cedars-Sinai Medical Center, Los Angeles, CA, USA; g Department of Pathology, University of Iowa, Iowa, IA, USA; h Department of Pathology, Aga Khan University, Nairobi, Kenya; i Department of Surgery, AGC Tenwek Hospital, Bomet, Kenya; j Department of Surgery, Brown University, Providence, RI, USA; k O'Neal Comprehensive Cancer Center, The University of Alabama at Birmingham, Birmingham, AL, USA

Dear Editor,

We respectfully submit the enclosed *Letter to the Editor* to appear alongside our full methodology paper, “Lessons Learned from the Validation of a Machine Learning-based Colorectal Carcinoma Screening Pipeline in Sub-Saharan Africa”. The dual purpose of this brief communication is to:

1. Highlight the broader methodological significance of the manuscript beyond its immediate technical results, and
2. Catalyze dialogue on how existing machine learning (ML) pipelines can be responsibly adapted to disparate patient populations and constrained lab environments.

## Why this contribution matters now

While many reports describe high-performance neural networks for histology, far fewer address the practical hurdles of transplanting those models into settings lacking robust compute, network bandwidth, or subspecialty expertise. Our study demonstrates that with careful modularization—and by packaging each stage into a self-contained Jupyter notebook—sites serving under-resourced communities can deploy state-of-the-art digital pathology within months rather than years.

**Fig. 1:**
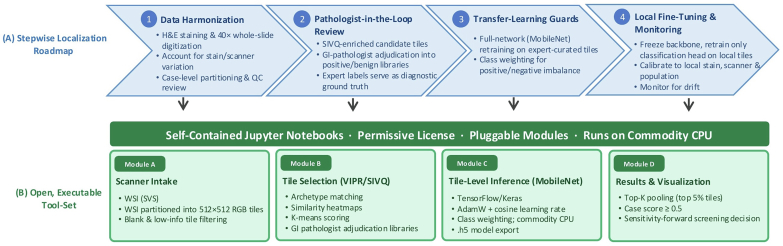
A best-practice framework and open toolset for localizing ML-based digital pathology in resource-constrained settings. The schematic illustrates a modular approach for adapting, validating, inspecting, and deploying machine-learning pathology pipelines paired with a permissively licensed, notebook-based toolset that packages each auditable workflow stage as a self-contained Jupyter notebook runnable on modest hardware and adaptable to local infrastructure, budgetary constraints, and regulatory requirements.

Two complementary dimensions (Fig. 1)

1. *Best-practice framework for localization.*
  We distill a stepwise roadmap (data harmonization, pathologist-in-the-loop review, transfer-learning guards, and local fine-tuning with monitoring). The mathematics in the supplementary materials formalizes key assumptions (e.g., the sensitivity-forward behavior of Top-K case-level pooling) so that readers may adapt thresholds to their own cohorts.
2. *Open, executable toolset.*
  The notebook suite—released under a permissive license—reproduces the entire workflow on modest hardware. By treating scanner intake, tile selection, tile-level inference, and results visualization as pluggable modules, labs can swap components as budgets or regulations dictate, without re-engineering the pipeline core.

## Call for community engagement

We invite fellow informatics teams to replicate, critique, and extend this framework. Specifically, we hope to stimulate comparative studies on:

• Latency and cost trade-offs when moving from on-premises GPUs to lightweight CPU inference.
• The impact of incremental domain adaptation when transferring models across diverse patient populations and lab settings.
• Governance strategies for continuous performance monitoring, where biostatistical support is limited.

The *Journal of Pathology Informatics* has long championed pragmatic, open solutions for global pathology challenges. By pairing this Letter with the full article, we believe the readership will gain both a strategic perspective on democratizing digital pathology and a concrete, immediately deployable toolkit.

Thank you for considering this submission. We look forward to contributing to the ongoing discussion within the JPI community.

Sincerely,

Alfred Githuka, Ulysses G.J. Balis, Ye Chan Kim, Eileen M. Weinheimer-Haus, Christopher L. Williams, Jerome I. Cheng, Kelou Yao, John Blau, Jessica A. Baker, Priscilla Njenga, Winny Chepkemoi, Robert K. Parker, Akbar K. Waljee, Shahin Sayed, and Mansoor N. Saleh

## Funding

Research reported in this publication was supported by the 10.13039/100000179Office Of The Director, 10.13039/100000002National Institutes Of Health (OD), the 10.13039/100000070National Institute Of Biomedical Imaging And Bioengineering (NIBIB), the 10.13039/100000025National Institute Of Mental Health (NIMH), and the 10.13039/100000061Fogarty International Center (FIC) of the 10.13039/100000002National Institutes of Health under award number U01CA287852. The content is solely the responsibility of the authors and does not necessarily represent the official views of the National Institutes of Health.

